# Lipid-Drug Conjugates and Nanoparticles for the Cutaneous Delivery of Cannabidiol

**DOI:** 10.3390/ijms23116165

**Published:** 2022-05-31

**Authors:** Aleksandra Zielińska, Amanda Cano, Tatiana Andreani, Carlos Martins-Gomes, Amélia M. Silva, Marlena Szalata, Ryszard Słomski, Eliana B. Souto

**Affiliations:** 1 Institute of Human Genetics, Polish Academy of Sciences, Strzeszyńska 32, 60-479 Poznań, Poland; ryszard.slomski@up.poznan.pl; 2 Department of Pharmacy, Pharmaceutical Technology and Physical Chemistry, Faculty of Pharmacy and Food Sciences, University of Barcelona, 08007 Barcelona, Spain; acanofernandez@ub.edu; 3 Institute of Nanoscience and Nanotechnology (IN2UB), 08028 Barcelona, Spain; 4 CIQ-UP—Chemistry Research Centre, Faculty of Sciences, University of Porto, 4169-007 Porto, Portugal; tatiana.andreani@fc.up.pt; 5 Centre for Research and Technology of Agro-Environmental and Biological Sciences (CITAB-UTAD), Quinta de Prados, 5001-801 Vila Real, Portugal; camgomes@utad.pt (C.M.-G.); amsilva@utad.pt (A.M.S.); 6 Department of Biology and Environment, University of Trás-os Montes e Alto Douro (UTAD), Quinta de Prados, 5001-801 Vila Real, Portugal; 7 Department of Biochemistry and Biotechnology, Poznań University of Life Sciences, Dojazd 11, 60-632 Poznań, Poland; szalata@up.poznan.pl; 8 Department of Pharmaceutical Technology, Faculty of Pharmacy, University of Porto, Rua de Jorge Viterbo Ferreira 228, 4050-313 Porto, Portugal; 9 UCIBIO/REQUIMTE, Faculty of Pharmacy, University of Porto, Rua de Jorge Viterbo Ferreira 228, 4050-313 Porto, Portugal

**Keywords:** lipid-drug conjugates, micelles, cutaneous drug delivery, cannabidiol, lipid nanoparticles

## Abstract

Lipid nanoparticles are currently used to deliver drugs to specific sites in the body, known as targeted therapy. Conjugates of lipids and drugs to produce drug-enriched phospholipid micelles have been proposed to increase the lipophilic character of drugs to overcome biological barriers. However, their applicability at the topical level is still minimal. Phospholipid micelles are amphiphilic colloidal systems of nanometric dimensions, composed of a lipophilic nucleus and a hydrophilic outer surface. They are currently used successfully as pharmaceutical vehicles for poorly water-soluble drugs. These micelles have high in vitro and in vivo stability and high biocompatibility. This review discusses the use of lipid-drug conjugates as biocompatible carriers for cutaneous application. This work provides a metadata analysis of publications concerning the conjugation of cannabidiol with lipids as a suitable approach and as a new delivery system for this drug.

## 1. Introduction

Nanotechnology has extensively been developed over the last decades, resulting in a diversity of nanosized drug delivery systems (DDS) for chemically different drugs and other bioactive ingredients, with relevance for academic research and for the pharmaceutical industry [1,2].

**Figure 1:**
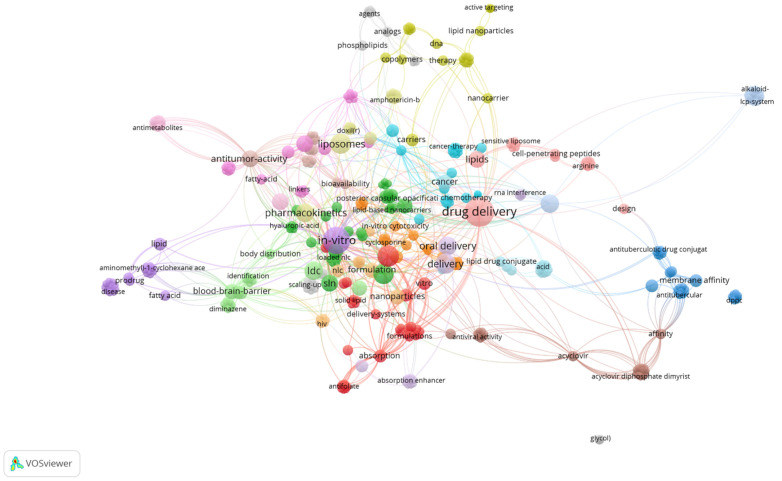
Bibliometric map obtained by VOSviewer software version 1.6.16 using “lipid drug conjugates” as the only keyword, from Web of Science database [3].

Nanosized DDS are aimed to (i) minimize the premature degradation of drugs after their administration; (ii) reduce the risk of systemic drug exposure and the development of undesirable side effects, and (iii) increase the bioavailability of drugs and the amount of a drug reaching the site of action. Drug delivery from DDS may be promoted by reactive stimuli occurring at the site of action, such as pH and temperature variations. On the other hand, it may also be desirable that DDS remains longer in the bloodstream and the drug is released in a controlled fashion, which can be achieved by means of developing lipid-drug conjugates (LDC). From a quick search in the Web of Science, a total of 2957 published works were exported. The bibliometric map obtained by VOSviewer software, using lipid-drug conjugates as the keyword is shown in Figure 1. Amongst the listed applications, LDC are mostly described for drug delivery aimed either for oral administration or for antitumor activity and to overcome blood brain barrier.

Site specific targeting can be accomplished by customizing nanoparticles with specific targeting moieties, including monoclonal antibodies [4,5,6,7]. In addition, it is desirable that the drug carrier remain in the bloodstream for long periods or as long as necessary to produce its desired therapeutic action. The prolonged circulation of drugs in the bloodstream allows for the maintenance of desired therapeutic levels for a longer time, thus prolonging the desired therapeutic action [8,9]. Moreover, a longer circulation allows drugs with high molecular weight molecules or drug-loaded microparticles to slowly accumulate in pathological sites with affected vasculature (such as tumors or inflammation, for example). Through an increased permeability and the retention effect that may exist in these locations, the concentrations of drugs in the target locations will increase [8,10,11,12,13,14]. The prolonged circulation also allows for an increase in the enhanced interaction between the drugs and the target organ, which is especially important for successful targeting in pathological areas with poor blood supply and/or a low concentration of drugs [11,12,15].

The development of carriers that have suitable characteristics such as (i) biocompatibility and biodegradability, (ii) adequate particle size, (iii) a high load capacity, (iv) prolonged circulation time, and (v) the ability to accumulate in pathological sites of action for adequate periods have been a challenge for the scientific community in the search for a system capable of solving and addressing deficiencies in the transport of hydrophilic drugs [16,17,18]. The availability of such vectors is essential since therapeutic applications with hydrophilic drugs are currently associated with some health problems. In this sense, it is necessary to emphasize that a low water solubility results in poor absorption and consequently a low bioavailability [19,20].

The formation of salts, or in some cases by adjusting the pH, facilitates the dissolution of drugs that are poorly soluble in water in case they present ionizable groups. In this sense, trying to overcome the low water solubility of some medications, some clinically accepted organic solvents have been used as adjuvants, as is the case with Cremephor^®^ EL (castor oil polyethoxylated) and surfactants [21,22,23,24,25,26]. However, the administration of some co-solvents and surfactants can cause toxicity or other undesirable side effects [27]. More recent approaches include liposomes, microemulsions, and cyclodextrins to increase the bioavailability of poorly water-soluble drugs [10,11,15,28]. However, despite showing promising results for certain poorly soluble drugs, liposomes and cyclodextrins also have a limited ability to incorporate hydrophilic drugs. Additionally, the solubilization capacity of these vectors varies for different drugs within extensive limits [11,15]. In this sense, a good alternative is the use of micelles of phospholipids and conjugates of lipids and medicines capable of carrying drugs of both hydrophilic and also lipophilic characteristics [11,13,14].

Cannabis has been used for medical purposes since 2700 BC. Additionally, in the nineteenth and early twentieth centuries, hemp was routinely used in Europe and in the United States. The abandonment of hemp-based products was associated with the introduction of synthetic drugs as well as international bans on the cultivation and marketing of cannabis. It is assumed that the detection and the characterization of pharmacologically active cannabinoids is associated with an increased interest in cannabis and its components [29,30]. There are three groups of cannabinoids: endocannabinoids produced in mammals, including humans; cannabis-produced phytocannabinoids; and synthetic cannabinoids. Concerning skin care, various health-promoting effects have been attributed to CBD, however most lack scientific validation or refer to the use of CBD-containing extracts or oils, which may also contain other cannabinoids. Table 1 shows recent publications regarding CBD bioactivities at skin level. As it can be observed, a wide variety of activities are already described, with an emphasis on the capacity to prevent UV-induced damage as UV radiation is often used in therapy against skin disorders such as psoriasis [31].

Endocannabinoids act through CB1 and CB2 receptors, and they affect processes related to memory, the brain’s reward system, analgesia, drug addiction, sleep regulation, mood, appetite, and metabolism. Among them, anandamide (AEA), palmitoylethanolamide (PEA), and noladim ether can be distinguished. In turn, the most studied are phytocannabinoids, which include about 120 out of over 560 active chemical ingredients found in *Cannabis sativa* L. plants. These include mainly delta 9-tetrahydrocannabinol with psychoactive properties, cannabidiol (CBD), cannabichromene, and cannabinol. Among synthetic cannabinoids, we can distinguish systemic phytocannabinoid analogues such as Dronabinol or Nabilone, as well as man-made substances, often with an effect many times greater than the psychoactive properties of THC [38,39].

Changes in therapeutic and recreational legislation allow the increasing use of cannabis and cannabinoids. Lipophilic properties and susceptibility to degradation limit the bioavailability of cannabinoids. Among the different delivery options for cannabinoids including oral, nasal inhalation, intranasal, mucosal (sublingual and buccal), transcutaneous (transdermal), local (topical), and parenteral, it seems that topical and transdermal products tend to have higher bioavailability, while limiting the psychotropic effects of the drug [30,40].

Cannabidiol (CBD) is a promising drug due to its broad spectrum of pharmacological actions. This *Cannabis sativa*-derived active ingredient has excellent therapeutic potential. Its development as an effective drug by the pharmaceutical industry is limited because of low bioavailability, low water solubility, and variable pharmacokinetics. Potential methods to overcome these limitations include drug delivery systems, improved crystal formulations, and other solid-state delivery formulations, mainly in the pre-clinical or early clinical stages of development [41]. In Table 2 are listed examples of advances in CBD-loaded lipid particles with a potential application for topical delivery of CBD.

Considering that cannabidiol has been recommended for skin disorders, such as eczema, psoriasis, pruritis, and inflammatory conditions [39,47,48], in this work we have evaluated the available literature that describe the use of lipid nanoparticles for the delivery of cannabidiol, with a special focus on cutaneous delivery. The Scopus database was used to search “solid lipid nanoparticles,” “cannabidiol,” and “cutaneous administration” as keywords and selecting published papers from 1990 until 2022. Duplicate articles found in more than one of the databases not focusing on selected keywords were excluded. The VOSviewer software version 1.6.16 (Leiden, The Netherlands) was used [3].

## 2. Lipid-Drug Conjugates

**Figure 2:**
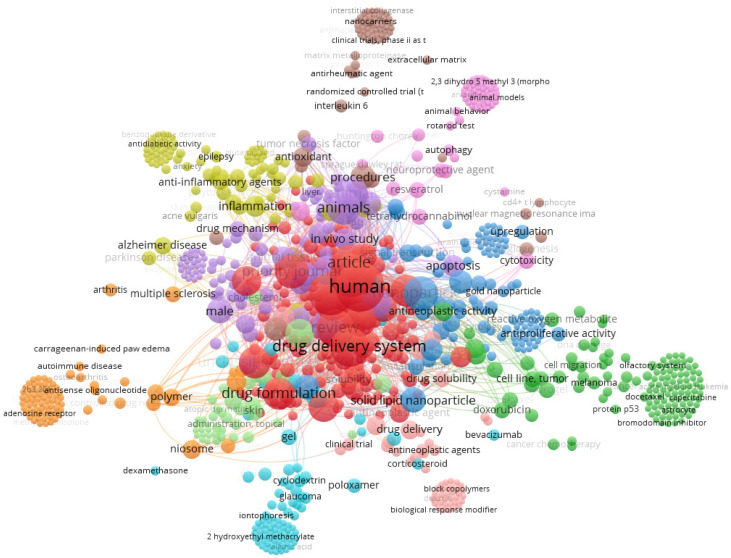
Bibliometric map obtained by VOSviewer software version 1.6.16 using solid lipid nanoparticles and cannabidiol as keywords, from Scopus database [3].

Lipid carriers are interesting for the transport of lipophilic drugs and, to some extent, hydrophilic drugs [18,49]. Some limitations of specific lipid carriers commonly investigated, such as nanoparticles produced only from solid lipids, and also nanostructured lipid carriers, include their low capacity to incorporate hydrophilic drugs due to the effects of stability during the production process. Only poorly water-soluble drugs can be conveniently incorporated into the solid lipophilic matrix. To overcome this limitation, lipid-drug conjugates have been developed, and up to the present loading capacities of up to 33% have been achieved [50,51]. The first search on cannabidiol and lipid nanoparticles resulted in the bibliometric map shown in Figure 2, showing the high amount of work done in the field of drug delivery and drug formulation for a range of applications (e.g., antidiabetic, anticancer, and anti-inflammatory actions, for multiple sclerosis and Alzheimer’s disease), including in clinical trials.

When compared to other lipid carriers (e.g., solid lipid nanoparticles and nanostructured lipid carriers), lipid-drug conjugates can exceed the minimal loading capacity for hydrophilic drugs, typically below 0.5% [52,53]. This is sufficient for highly potent peptides and proteins, such as eosinophil peroxidase (EPO) and interferons, as these can be solubilized in the fused lipid matrix, using mixtures of surfactants or acyl and diacylglycerols present in the lipid [49]. Lipid-drug conjugates have numerous advantages, namely: (i) protection against enzymatic and chemical degradation of drugs [50]; (ii) an increase in the lipophilicity of the hydrophilic compound [49,50,54]; (iii) easier passage through the blood–brain barrier [50]; (iv) an increase in the load capacity for hydrophilic drugs [49,50,54]; (v) increased stability in vivo, reducing solubility and subsequently accessibility by enzymes [49,51]; and (vi) an improvement in the permeability of the lipophilic molecule across membranes [51,55].

**Figure 3:**
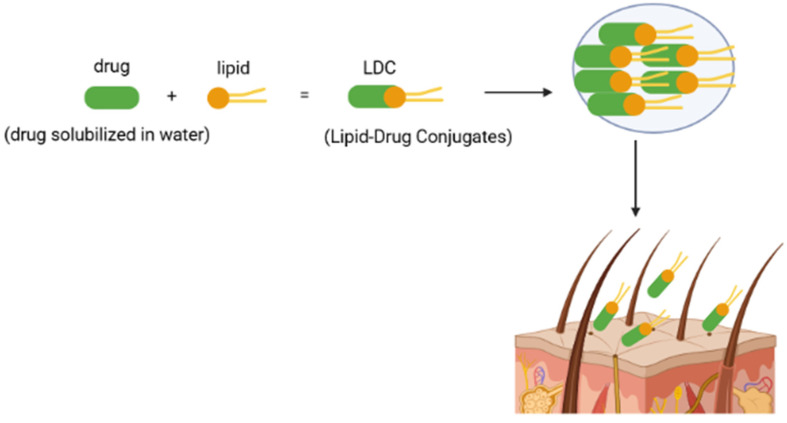
Schematic representation of the lipid-drug conjugates mechanism of action [own drawing].

The principle of lipid-drug conjugates lies in transforming drugs with hydrophilic characteristics into a more lipophilic and consequently a more insoluble molecule by conjugation with a lipidic compound (Figure 3) [49]. Thus, the conjugation can be carried out by forming a salt with a fatty acid, for example, the reaction of the functional groups of the drugs with the carboxylic groups of the fatty acids [51,54] or alternatively by covalent bonding (for example, ether, ester) [49,51].

**Figure 4:**
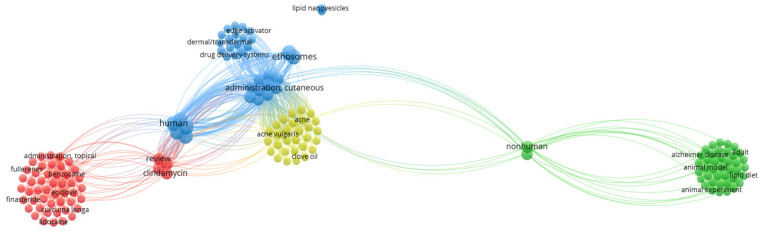
Bibliometric map obtained by VOSviewer software version 1.6.16 using cannabidiol and cutaneous administration as keywords, from the Scopus database [3].

Obtaining the formulation of cannabidiol-loaded lipid nanoparticles is difficult because of the low water solubility of CBD. However, several studies have already proven the successful and efficient production of CBD-high-loaded lipid carriers for parenteral or oral application. Lipid carriers allow parenteral administration in therapy with CBD, which could be of interest due to the broad field of pharmacological effects. Francke et al., (2021) [43] have shown that a higher drug loading in emulsions and self-dispersing mixtures than in liposomes emphasizes an advantage of oil-containing carrier systems for CBD formulation. For oral therapy, phospholipids as natural emulsifiers and solubilizers can generate self-dispersing lipid formulations. Due to mild agitation, these formulations can be filled into hard capsules and dispersed in gastrointestinal fluids [43]. Figure 4 shows that cannabidiol has been reported for skin infection and the treatment of acne vulgaris, in combination with other synthetic drugs, and also formulated as lipid nanoparticles to improve its bioavailability.

**Figure 5:**
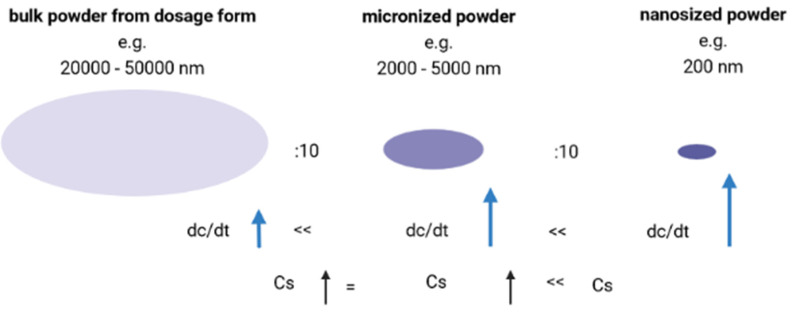
Schematic representation of the variation in the dissolution speed of saturation solubility as a function of particle size [own drawing].

The poor bioavailability of drugs, when administered topically, is mainly due to two reasons: (i) low dissolution rate and (ii) poor permeability [16,49,51,54,56]. This can be explained by the Noyes–Whitney Equation, which describes the dissolution rate (*dc*/*dt*), which is proportional to the concentration gradient (*cs* − *cx*/*h*), where the cs factor is the total concentration of drugs in the liquid (solubility saturation). The *h* factor is the diffusion distance above the surface of the drugs particle. The dissolution rate is given as a function of the surface area:

$$\frac{dc}{dt}=\frac{DA\;\left(cs-cx\right)}{h}$$

where *D* is the diffusion coefficient and *A* is the surface area. The higher the stirring speed in the dissolving medium, the lower the value of *h* and, consequently, the higher the dissolving rate of the drugs. The ideal situation is when *h* tends to zero and, simultaneously, *dc*/*dt* tends to infinity (Figure 5) [57,58].

To obtain lipid-drug conjugates, drugs are added to an aqueous solution with an excess of sodium hydroxide. Subsequently, a precipitate forms, which is filtered through a filter paper and then washed three times with water suitable for injections. Then, it is dried at 40 °C and stored at 4 °C. The fatty acid salts of drugs are prepared by dissolving this and the fatty acids (e.g., oleic acid and stearic acid) in an appropriate solvent and consequent solvent evaporation under reduced pressure. The formulations obtained, i.e., the conjugates, are dried for 24 h [54,59]. Another method of obtaining lipid-drug conjugates includes high-pressure homogenization. Fatty acid vesicles with drugs are prepared by dispersing the residue in a surfactant (i.e., Tween^®^80) [50,54] containing glycerol, forming a pre-dispersion. This pre-dispersion is subject to homogenization at high pressure, comprising 20 to 27 cycles at a pressure of 1500 bar. Some authors [50] argue that after five cycles, the samples should be placed on ice to avoid the high temperatures—temperatures well above 40 °C—of the dispersion.

Lipid-drug conjugates have been widely used for brain therapy, specifically in treating sleeping sickness caused by *Trypanosoma brucei gambiense* [50,54,59]. Currently, no products are available that use this technology for cutaneous application. However, this methodology presents a therapeutic advance, a new approach to transport hydrophilic drugs in a lipophilic medium, as is the case of the skin structure. Some factors that may justify the non-exploitation of this methodology at the top level may be:

- The traditional pharmaceutical formulas (e.g., creams and ointments) have responded to different pathologies as much as possible;
- Unlike many other structures and organs, the skin’s composition allows a broad absorption of hydrophilic and lipophilic drugs;
- Simply the development of other transport mechanisms, such as the phospholipid micelles.

However, if developed for a cutaneous application, this therapeutic approach would present numerous advantages: (i) bioavailability; (ii) the use of biocompatible lipids, which would lead to more significant bioavailability; (iii) the ability to deliver hydrophilic drugs to more lipophilic compartments of the skin’s constitution; (iv) a decrease in the number of drugs used; and (v) more excellent protection for drugs from degradation agents. Therefore, this therapeutic approach presents several relevant and promising aspects for a future, possible cutaneous application [49,55,56].

**Figure 6:**
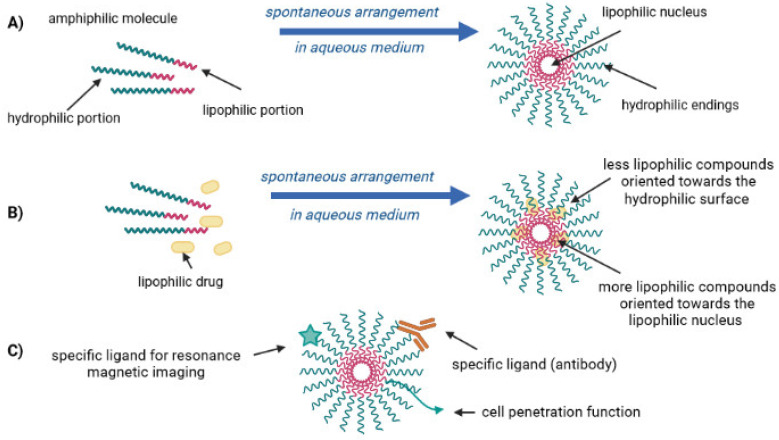
Schematic representation of pharmaceutical micelles [own drawing]. (**A**) spontaneous formation of a micelle, of an amphiphilic molecule in an aqueous medium; (**B**) incorporation of lipophilic drugs into a micelle; (**C**) multifunctional pharmaceutical micelles [own drawing].

Micelles are colloidal dispersions (i.e., vesicles of size comprised between 10–100 nm) of amphiphilic nature; their composition is simultaneously a lipophilic portion (nucleus) and a hydrophilic portion (outer surface). These vesicles are currently used to transport lipophilic drugs, among other functionalities (Figure 6).

Micellar technology was introduced in the last decade of the 20th century [60]. Subsequently, scientists developed Novavax^®^, patenting micelle technology and, later, launched the first nano-product of transdermal lotion (Estrasorb^TM^) in 2003 [48]. Compounds used in Estrasorb^TM^ are generally recognized as safe (GRAS) as micellar vesicles are formulated based on nanotechnology that achieves an advance in transdermal therapy. The formulation represents a robust and a versatile delivery system, accommodating a range of therapeutic composts with different physical–chemical properties [60,61,62,63,64,65]. As micellar vesicles, in the form of emulsions (lotions), are attractive alternatives for delivering bioactives through topical application. The technology allows high concentrations of drugs to penetrate the skin, and it functionally creates a deposit of drugs in the stratum corneum and epidermis, avoiding the first passage effect [11]. These two formulations could be exploited for the transdermal delivery of cannabidiol.

**Figure 7:**
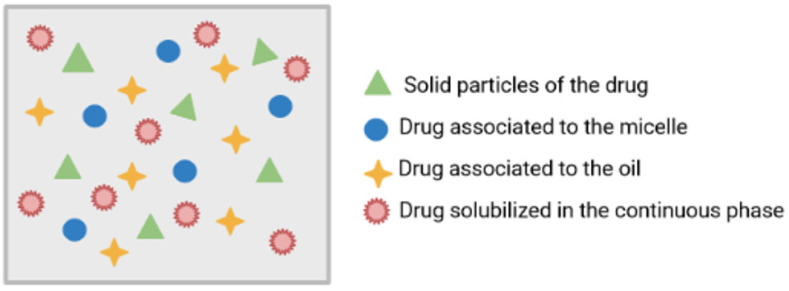
Schematic representation of the formation of phospholipid micelles, showing the different behaviors of drugs [own drawing].

Due to the formation of micelles and driven by the reduction of free energy in the system, the removal of two hydrophobic fragments from the aqueous environment consequently results in a network of hydrogen bonds in water. The hydrophobic fragments of the amphiphilic molecule form the nucleus of a micelle, while the hydrophilic components constitute the outer surface of the micelle [11,15]. In general terms, in the formation of phospholipid micelles, the formation of a multiphase nanoemulsion occurs (hydrophilic and lipophilic portion simultaneously). In this sense, there are five essential components for the construction of phospholipid micelles, namely (i) drugs; (ii) the solvent; (iii) a surfactant; (iv) phospholipid, and (v) the aqueous medium. When these compounds are added and subjected to a homogeneous mixing process, the drugs can have one or more conformations (Figure 7): (a) solid particles; (b) drugs associated with the micelle; (c) drugs associated with the oil; and/or (d) solubilized (micro/nanoparticles).

Although micellar technology can preferentially accommodate crystalline compounds, surprisingly, it can also be used for amorphous compounds. The formed micellar system can also accommodate poorly soluble drugs. Depending on the physical–chemical properties of the drugs and the dosage requirements, the load capacity that can be achieved through this system is up to 20% [66,67].

The solvent is generally used to assist solubilization of the drugs during processing, although it is not a prerequisite. The typical solvent used in the preparation of phospholipid micelles is ethanol. In addition, stable micelles can be obtained through other solvents, such as propylene glycol, low molecular weight polyethylene glycol, triacetin, and N-methyl pyrrolidinone. The solvent plays an essential role in controlling the solubilized fraction of drugs, critical for facilitating drug permeability. The stabilizers used are generally non-ionic. Stable micelle preparations have been prepared using hydrophilic and lipophilic stabilizers that encompass a range of products with an appropriate hydrophilic-lipophilic balance (HLB) [68]. Surfactants include sorbitan esters, glycerol esters, block copolymers, polyethylene glycol esters, and ethoxylated fatty esters. Surfactants help stereochemically stabilize oil droplets and contribute to the formation of the micellar phase [11,14,15,66]. Table 3 describes some phospholipids used to form micelles and the final particle size. The lipids are from the internal phase of the micelle. Depending on the properties of the drugs, the lipophilic phase can accommodate a fraction of the drug’s insoluble form. The aqueous medium used is generally purified water. A buffering agent can be included to maintain the pH and to maximize the stability of the drugs. The phospholipid micelles are dependent on therapeutic need as well as the physical–chemical properties of the drugs, the intended site of action (local or systemic), and the profile of the target product. For topical or transdermal administration, the micellar system can be classified as a type of micro-reservoir system of controlled dissolution that can be adapted for the release of drugs topically (where the site of action is the skin) or transdermally (systemic availability). The physical–chemical properties of the formulation can be adapted, depending on the type of administration intended [69].

The composition of a phospholipid micelle formulation is inherently antimicrobial. According to the results of the United States Pharmacopeia (USP) antimicrobial efficacy tests for a placebo formulation of phospholipid micelles, it indicates that the micellar formulation not only impedes bacterial growth but essentially presents microbicide activity. This can be attributed to the small size of the preparation (in the order of nanometers) and the nature of the composition (that is, the high concentration of non-ionic surfactant). However, a micellar formulation exhibits a good safety profile, and it is relatively dermatologically a non-irritant [11]. This property offers commercial advantages such as the possible elimination of an antimicrobial preservative (especially for a product packaged in a multidose container), or the possible synergy of the microbicidal effect of a micellar preparation when prepared with an antibacterial, antifungal, antiviral agent [11].

Transdermal administration involves the application of a pharmacologically active compound on the skin to reach the systemic circulation to reach the site of action, often away from the site of application. Since the approval of the first transdermal delivery system for drugs in 1981, *Transderm-Scop*^®^, there has been intense research in the field of transdermal therapy for the treatment of a variety of clinical conditions [70]. Transdermal administration is particularly advantageous for drugs with a significant hepatic first-pass effect or degradation in the gastrointestinal tract.

Although lately there has been a good development of formulations at the transdermal level, there is still a low point of innovation about the vectorization and delivery of drugs at the top level [66,67]. Most dosage forms are limited to traditional creams, ointments, and gels. Some of the new commercial applications are sprays and foams. Phospholipid micelle technology can be exploited to create better dosage forms at the top level, guaranteeing efficient and effective delivery of drugs locally (at the application site). It is possible to adapt a drugs deposition, disposition, and permeability kinetics through formulation engineering (altered composition, drug charge, particle size, among others). The ever-growing interest in CBD’s health-promoting bioactivities and the benefits of lipid nanoparticles and lipid particles as delivery systems to overcome the low bioavailability of CBD has advanced in recent years. This led to the registration of various patented works for applications of CBD for skin care as well as formulations to deliver the active ingredient. In Table 4 we present several examples of patents within this scope.

## 3. Conclusions

Transport and the transdermal delivery system for compounds are not suitable or clinically justified for all drugs. Therefore, they are often seen as a very restricted and more limited mechanism than they are. Due to the high number of bioactivities described for CBD, new delivery systems that improved drug stability and bioavailability were mandatory. At the same time, CBD benefits on skin have been reported, thus this organ arising as a focus for CBD delivery. Micellar phospholipid technology helps to integrate and to deliver many therapeutic compounds, which are otherwise seen as unsuitable for transdermal administration. This technology allows for rapid and low-cost development, compared to the typical story of new chemical entities. The data from the preclinical studies described here show a high probability of clinical success, in addition to demonstrating advantages both in the context of a shorter period of development of the formulation or at a lower cost than the development of a traditional pharmaceutical form. Understanding the basic physico–chemical properties of micellar formulations allows greater control and manipulation over pharmacokinetic parameters, providing an attractive option for pharmaceutical technology and, consequently, for the treatment of many pathologies. The technology is validated for the transdermal delivery of compounds, and the commercial product, Estrasorb^TM^, is manufactured on a significant scale. Estrasorb^TM^’s ingredients are GRAS, and the manufacturing process is attractive from a cost perspective. The multiphase nanoemulsion that comprises the micellar formulation is surprisingly stable and, in some cases, subject to terminal heat sterilization. The scientific community, at present, has dedicated new studies to the development of new pharmaceutical forms, according to micellar technology, particularly for compounds that are poorly soluble in water. It should be noted that experiments have demonstrated the potential of micellar technology to be used for the topical route, being just a matter of time for the emergence of new pharmaceutical forms based on the micellar technology of phospholipids.

## Figures and Tables

**Table 1:** Health-promoting bioactivities of CBD at skin level.

CBD Formulation	Experimental Model	Bioactivity	Ref.
2.5%	Rat	Prevent protein modulation by UVA and UVB exposure	[31]
2.5%	Rat	Protection against UV-induced damage	[32]
10 µM	Human keratinocyte cell model (HaCaT)	Protection against H_2_O_2_- induced oxidative damage	[33]
1%	Mice	Moisturizing activity	[34]
0.04–0.2 mg/mL	Mice melanoma cell line (B16-F)	Anti-proliferative activity	[35]
n.s.	Human clinical case studies	Treatment of Epidermolysis bullosa	[36]
10 µM	Human immortalized SZ95 sebocytes	Treatment of acne vulgaris Anti-inflammatory activity Sebostatic activity	[37]

Notes: n.s.—not specified.

**Table 2:** Examples CBD-loaded lipid particles with potential for skin application.

Formulation	Composition	Size	Application	Ref.
Ethosome	CBD, EtOH, Phospholipon 90	300–400 nm	Increased skin permeation Anti-inflammatory activity	[42]
Emulsion	CBD, Oil phase (Soybean oil, rapeseed oil, Trimyristin or Miglyol 812), Poloxamer (188 or 407), Sodium azide	69–233 nm	Increased drug loading	[43]
Emulsion	CBD, chitosan, collagen, oil phase (olive oil or liquid paraffin)	n.s.	Increased delivery and deposition in the *stratum corneum*	[44]
Emulsion	CBD, chitosan (various deacetylation degrees), gum Arabic, olive oil	45–787 nm	Higher skin absorption	[45]
Emulsion	CBD, isopropyl myristate, Solutol HS 15 and Transcutol P	35 nm	Development of a microemulgel for the treatment of skin disorders	[46]

Notes: n.s.—not specified.

**Table 3:** Phospholipids used in the formation of micelles and the final particle size.

Micelles	Mean Vesicle Size (mm)
PEG750-DSPE	7–15
PEG2000-DSPE	7–20
PEG5000-DSPE	10–40
PEG2000-DOPE	7–20
PEG5000-DOPE	10–35
PVP1500-P	5–15
PVP8000-P	7–20
PVP15000-P	-
PVP1500-S	5–15
PVP8000-S	10–20
PVP15000-S	-

Captions: PEG, Polyethylene glycol; PVP, Poly (N-vinyl -2-pyrrolidone); DOPE, Dioleolylphosphatidylethanolamine; DSPE, Phosphatidylethanolamine distearate; P, Palmitoil; S, Stearyl.

**Table 4:** Examples of patented formulations aiming CBD use in skin care products or in lipidic particle formulations.

Patent	Application
CA2760460C	CBD transdermal formulation with enhanced penetration to be used in inflammation and pain treatment
WO2019244160A1	Anti-microbial hyperosmotic formulation containing CBD
USRE47885E1	CBD-containing hydrogel developed for transdermal (microneedle) or topical application
US11260033B2	CBD-loaded lipid nanoparticles for increased stability and bioavailability
US8435556B2	Transdermal formulation containing CBD and diethylene glycol monoethyl ether as penetration enhancer
US20210244680A1	Wearable transdermal patch with CBD-loaded liposomes
BR112020003025A2	Transdermal gel containing CBD for osteoarthritis treatment
US10842758B1	Transdermal delivery formulation containing CBD, phosphatidylcholine, safflower oil, oleic acid, stearic acid, and isopropyl palmitate

## Data Availability

Not applicable.
